# A randomized trial of dihydroartemisinin–piperaquine versus artemether–lumefantrine for treatment of uncomplicated *Plasmodium falciparum* malaria in Mali

**DOI:** 10.1186/s12936-018-2496-x

**Published:** 2018-10-05

**Authors:** Souleymane Dama, Hamidou Niangaly, Moussa Djimde, Issaka Sagara, Cheick Oumar Guindo, Amatigue Zeguime, Antoine Dara, Abdoulaye A. Djimde, Ogobara K. Doumbo

**Affiliations:** Malaria Research and Training Centre, Faculty of Pharmacy and Faculty of Medicine and Dentistry, University of Sciences, Techniques and Technology of Bamako, P.O. Box 1805, Bamako, Mali

**Keywords:** Dihydroartemisinin, Piperaquine, Artemether, Lumefantrine, In vivo

## Abstract

**Background:**

Artemether–lumefantrine (AL) and artesunate–amodiaquine are first-line treatment for uncomplicated malaria in many endemic countries, including Mali. Dihydroartemisinin–piperaquine (DHA–PQ) is also an alternative first-line artemisinin-based combination therapy, but only few data are available on DHA–PQ efficacy in sub-Saharan Africa. The main aim of this study was to compare clinical efficacy of DHA–PQ versus AL, using the World Health Organization (WHO) 42-day in vivo protocol.

**Methods:**

The efficacy of three-dose regimens of DHA–PQ was compared to AL combination in a randomized, comparative open label trial using the WHO 42-day follow-up protocol from 2013 to 2015 in Doneguebougou and Torodo, Mali. The primary endpoint was to access the PCR-corrected Adequate Clinical and Parasitological Responses at day 28.

**Results:**

A total of 317 uncomplicated malaria patients were enrolled, with 159 in DHA–PQ arm and 158 in AL arm. The parasite positivity rate decreased from 68.4% (95% CI 60.5–75.5) on day 1 to 3.8% (95% CI 1.4–8.1) on day 2 for DHA–PQ and 79.8% (95% CI 72.3–85.7) on day 1 to 9.5% (95% CI 5.4–15.2) on day 2 for AL, (p = 0.04). There was a significant difference in the uncorrected ACPR between DHA–PQ and AL, both at 28-day and 42-day follow-up with 97.4% (95% CI 93.5–99.3) in DHA–PQ vs 84.5% (95% CI 77.8–89.8) in AL (p < 0.001) and 94.2% (95% CI 89.3–97.3) in DHA–PQ vs 73.4% (95% CI 65.7–80.2) in AL, respectively (p < 0.001). After molecular correction, there was no significant difference in ACPRc between DHA–PQ and AL, both at the 28-day and 42-day follow-up with 99.4% (95% CI 96.5–100) in DHA–PQ versus 98.1% (95% CI 94.5–99.6) in AL (p = 0.3) and 99.3% (95% CI 96.5–100) in DHA–PQ vs 97.4% (95% CI 93.5–99.3) in AL (p = 0.2). There was no significant difference between DHA–PQ and AL in QTc prolongation 12.1% vs 7%, respectively (p = 0.4).

**Conclusion:**

The results showed that dihydroartemisinin–piperaquine and artemether–lumefantrine were clinically efficacious on *Plasmodium falciparum* parasites in Mali.

## Background

The emergence and spread of resistant *Plasmodium* strains to classic monotherapy drugs led the World Health Organization (WHO) to recommend artemisinin-based combination therapy (ACT) for uncomplicated malaria in 2006 [1]. By combining two active ingredients with different mechanisms of action, ACT is the most effective anti-malarial medicine available today. Since 2006, two ACT formulations, artemether–lumefantrine (AL) and artesunate–amodiaquine (ASAQ), were chosen by the National Malaria Control Programme to treat uncomplicated malaria in Mali. Many studies showed that AL and ASAQ remained efficacious against uncomplicated falciparum malaria in Africa [1–3] and data on clinical efficacy, safety and tolerability of AL and ASAQ exists in Mali [4, 5]. Dihydroartemisinin–piperaquine (DHA–PQ) is also recommended by the WHO for uncomplicated malaria in Africa but, few data are available on its clinical efficacy and safety [6, 7] but little is know on DHA–PQ combination. DHA–PQ is a potential alternative for the treatment of uncomplicated malaria in Mali. Therefore, clinical efficacy and safety data on DHA–PQ are required before its use in Mali as recommended by the WHO as an alternative choice. The aim of this study was to evaluate the in vivo efficacy and tolerability of the DHA–PQ compared to AL in the treatment of uncomplicated malaria in Mali.

## Methods

### Study site

The study was carried-out in two malaria endemic villages Doneguebougou and Torodo, two nearby villages, Mali. Doneguebougou and Torodo are located at 30 km and 37 km respectively in the North East of Bamako with a population of approximately 2500 and 2000 inhabitants respectively in north Savana zone. Malaria transmission is highly seasonal, occurring during the raining season. Entomologic inoculation rates were more than 100 infective bites per person per year.

### Study population

Patients with uncomplicated malaria were screened and allocated in the study arms at the community health centre from November 2013 to December 2015. Inclusion criteria were: older than or equal to 6 months, with an axillary temperature of ≥ 37 °C or history of fever within 24 h prior enrollment, being resident of Doneguebougou and Torodo since 6 months, able to take oral treatment, a parasitaemia between 1000 and 100,000 asexual *Plasmodium falciparum*/μl of blood, having QTc ≤ 450 ms, to sign an informed written consent and to have not used any ACT component within 28 days before enrollment. Patients were excluded if they had symptoms or signs of severe malaria, were unable to take oral medication, had an allergy to one of the study drugs or were pregnant (detected with a urine beta-human chorionic gonadotropin test). The protocol was reviewed and approved by the ethical committee of the Faculty of Medicine, Pharmacy and Odonto-stomatology, University of Sciences, Techniques and Technology of Bamako (N°2012/89/CE/FMPOS 20 December 2012).

### Treatment arms and follow-up

Enrolled patients were randomly assigned to receive either DHA–PQ (Malacur^®^, SALVAT, S.A., Spain) or AL (Coartem^®^; Novartis). There were two formulations of DHA–PQ, both were fixed-dose drug. The first formulation was a blister of tablets, each containing 40 mg of dihydroartemisinin (DHA) and 320 mg of piperaquine (PQ). The second formulation was an oral suspension of 90 mg of DHA and 720 mg of PQ in 60 ml solution (for children). AL was a fixed-dose combination tablets, each containing 20 mg of artemether and 120 mg of lumefantrine. DHA–PQ was administrated according to body weight (10–19.9 kg: one tablet; 20–39.9 kg: two tablets; ≥ 40 kg: three tablets). DHA–PQ suspension was administrated to body weight (3.5–5 kg: 5 ml; 6–9 kg: 10 ml; 10–12 kg: 15 ml; 13–17 kg: 20 ml). AL was administrated according to body weight (5–14 kg: one tablet; 15–24 kg: two tablets; 25–34 kg: three tablets; ≥ 35 kg: four tablets).

Dihydroartemisinin is the active metabolite of the artemisinin derivatives. It is effective against *P. falciparum* multidrug-resistant. Piperaquine is a bisquinoline anti-malarial drug that was first synthesized in the 1960s, and used extensively in China and Indochina as prophylaxis and treatment during the following 20 years [8]. Artemether is a methyl ether of dihydroartemisinin effective against *P. falciparum*. The injectable form is specifically used for severe malaria management. It is administered in combination with lumefantrine for improved efficacy and probably reducing resistant parasites selection against both drugs. Lumefantrine is an arylaminoalcohol, its action is to inhibit the polymerization of haem (toxic to the parasite) in malaria pigment. All treatments were administered orally in direct observation by a clinician of the research team at the health centre. In case of vomiting within 30 min of dose ingestion, a second full dose was given; if vomiting occurs in 1 h after dose ingestion a half of dose was administred.

Randomization codes were generated and sealed in individual opaque and sequentially numbered envelopes by the study statistician, who was not involved in the participant’s enrolment or outcome assessment procedures. The enrolled participants were assigned a treatment according to the envelope content. Thereafter, they were followed actively and passively for 42 days following a standard protocol [9].

Haemoglobin level was measured using a Hemocue^®^ apparatus, and anaemia was defined haemoglobin value < 10 g/dl. Thick and thin blood smears were made on the same slide and examined for asexual and sexual parasites positivity after staining in 10% Giemsa. Slides were also prepared on follow-up days 1, 2, 3, 7, 14, 21, 28, 35, and 42. Blood smears were read by trained readers according to WHO standard protocol. Slides readers were kept blinded to the treatment arm until the end of the study; this was conducted to minimize assessment bias because malaria parasite count was the key outcome used to define treatment failure.

A 12-lead electrocardiogram (ECG) study was performed on each volunteer prior to the first dose (at 0 h) of the study drug and at day 2 (4–5 h after the last dose). QTc in resting ECG was obtained using Bazett’s formula (QTcB) and Fridericia formula (QTcF). The ECG was taken over at least on day 3 if abnormal on day 2. Abnormal means that the QTcB or QTcF > 450 ms or the increase compared to the basic trace > 30 ms. An ECG monitoring system by a qualified investigator was set up at the site to monitor the quality of the ECG measurements during the study.

### Data collection and classification of therapeutic responses

The data was recorded on each day of follow-up. The case report form was used to record the patients’ study number, case history, study drugs, and all the clinical and parasitological data from day 0 to day 42, and the final classification of therapeutic response. The outcomes were classified in three categories responses, namely Early Treatment Failure (ETF), Late Treatment Failure (LTF) and Adequate Clinical and Parasitological Response (ACPR) according WHO protocol.

### Molecular correction

For participants with recurrent parasitaemia after day 7, paired Dried Blood Spots (DBS) from day 0 and the day of parasite recurrence were analysed for parasite merozoite surface protein 2 genes (*msp2*) and microsatellite (CA1 and TA87), to discriminate re-infection from recrudescence as described previously [10, 11].

### Power and sample size calculation

The sample size calculation was based on the non-inferiority hypothesis testing for the primary endpoint (proportion of PCR corrected parasitological and clinical cure rate at day 28 after for DHA–PQ compared to standard treatment drugs in Mali, that is AL). Based on previous study, the hypothesis for the reference treatment cure rate was 95% [12]. Non-inferiority delta was estimate at 7%, with a two-sided significance level α of 5% and power of 80%. On the basis of the above assumption, 151 patients were necessary per treatment arm, totaling 302 patients for the two treatment arms. This is the minimum number of patients to be included in order to conclude on the primary endpoint. By setting non-evaluability rate at 5%, a total size needed for this study was 317 patients.

### Statistical analysis

Baseline descriptive statistics were expressed as means or proportions. Categorical variables were compared by Chi square test or Fisher’s exact test. Continuous variables were compared using two sample t-test or Mann–Whitney test as appropriate. All statistical analyses were done with Stata version 11.0 (Stata Corp., College Station, TX). A p-value < 0.05 (two sided) was considered statistically significant.

## Results

### Participants’ characteristics

**Fig. 1:**
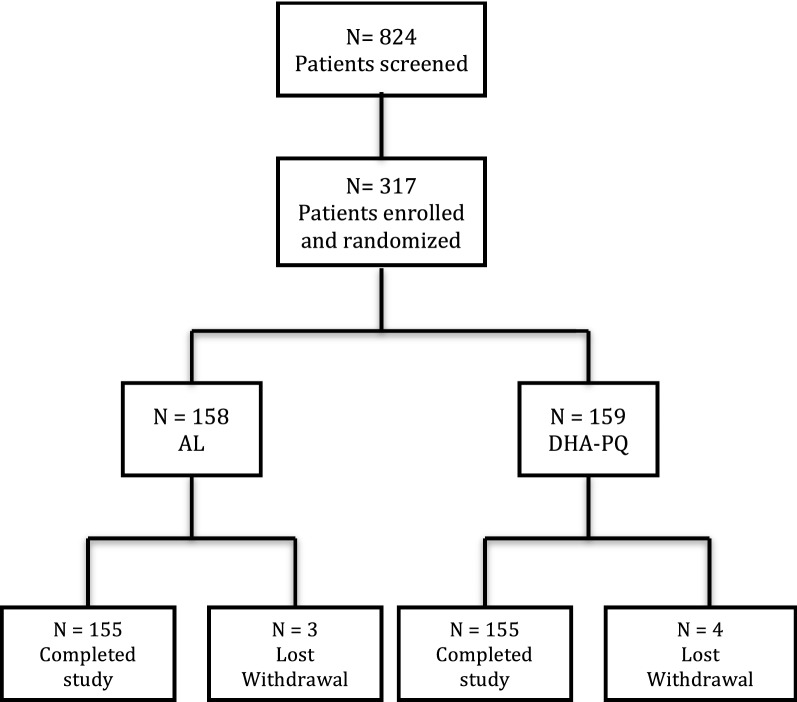
Trial profile

Three hundred seventeen of the 824-screened patients were enrolled as shown in Fig. 1. One hundred fifty-nine and 158 were randomized to receive DHA–PQ and AL, respectively. Of the 317 participants, 7 (2.2%) were lost of follow-up (one of the seven participant died after taking one dose of AL. Baseline characteristics of participants were similar at enrollment in the two study arms as shown in Table 1.

**Table 1:** Demographic, clinical, and laboratory characteristics of patients at baseline

Characteristics	AL (n = 155)	DHA–PQ (n = 155)	p values
Age group, years			
% < 5 years of age	21	23	0.6
% 5–53 years of age	79	77	
Gender categories			
% of female	47	46	0.6
% of male	53	54	
% of fever	67	69	0.7
% of anemia	42.7	41.1	0.7
Median of parasitemia (IQR 25th and 75th percentiles) Number of trophozoite/μl of blood	24,325 (9450–45,775)	21,200 (9280–38,875)	0.2

### Cure rates

Parasite positivity rate from day 1 to day 3 was calculated. The overall parasite clearance rate was rapid for two study drugs. The parasite positivity rate (PPR) decreased from 68.4% (95% CI 60.5–75.5) on day 1 to 3.8% (95% CI 1.4–8.1) on day 2 for DHA–PQ and from 79.8% (95% CI 72.3–85.7) on day 1 to 9.5% (95% CI 5.4–15.2) on day 2 for AL, (p = 0.04), from day 2 to 0.6% (95% CI 0.02–3.5) on day 3 for DHA–PQ and from day 2 to 0 for AL, (p = 0.3).

**Fig. 2:**
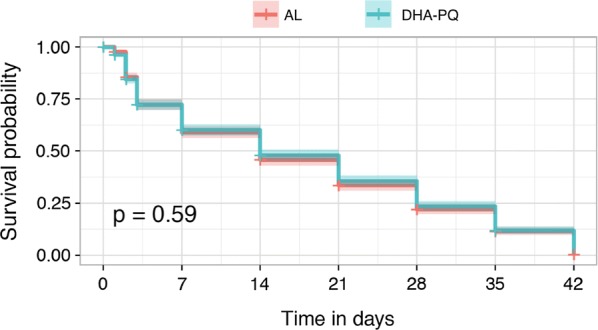
Kaplan–Meier of survival curves to show the probability of survival between treatment arms during follow-up

The per protocol analysis 28-day ACPR without PCR correction were 97.4% (95% CI 93.5–99.3) in DHA–PQ versus 84.5% (95% CI 77.8–89.8) in AL, respectively. Without PCR correction, DHA–PQ ACPR was statistically higher than AL at 28-day follow-up (p < 0.001) as shown in Table 2. Forty-two-day follow-up shown that DHA–PQ had a higher cure rate 94.2% (95% CI 89.3–97.3) than AL cure rate 73.4% (95% CI 65.7–80.2) without molecular correction as shown in Table 3 (p < 0.001). After PCR adjusting for cases of reinfection, the 28-day and 42-day follow-up cure rates were: 99.4% (95% CI 96.5–100) in DHA–PQ versus 98.1% (95% CI 94.5–99.6) in AL and 99.3% (95% CI 96.5–100) in DHA–PQ versus 97.4% (95% CI 93.5–99.3) in AL as shown in Tables 2 and 3. This difference between treatment arms was no longer significant (p > 0.05). Episodes of recurrent parasitaemia were first detected 14 days after treatment initiation both in the AL arm and DHA–PQ arm. Reinfection rates among participants who achieved PCR-adjusted parasitological cure by day 28 were 13.5% (95% CI 8.12–18.9) for the AL group and 0.6% (95% CI 0.01–3.5) for DHA–PQ, showing a significantly lower risk of reinfection after DHA–PQ treatment compared to AL [risk difference (RD): 12.9% (95% CI 7.38–18.4) unadjusted; p < 0.001]. Kaplan–Meier of survival curves showed that there were no differences (p = 0.59) in cure rates between two-study treatment arms as showed in Fig. 2. Reinfection rates among participants who achieved PCR-adjusted parasitological cure by day 42 were 24% (95% CI 17.2–30.7) for the AL group and 5% (95% CI 1.6–8.4) for DHA–PQ group. The risk difference had shown a significantly lower risk of reinfection after treatment with DHA–PQ, RD 19% (11.4–26.6, p < 0.001).

**Table 2:** Clinical and parasitological response at 28-day before and after PCR correction

Treatment arm outcome	AL n (%)	DHA–PQ n (%)	p-value
Before PCR correction			
ETF	0 (0.00)	1 (0.7)	1
LCF	8 (5.2)	1 (0.7)	0.036
LPF	16 (10.3)	2 (1.2)	0.001
ACPR^a^	131 (84.5)	151 (97.4)	< 0.001
Total	155 (100)	155 (100)	
After PCR correction			
ETF	0 (0.00)	1 (0.7)	1
LCF	0 (0.00)	0 (0.00)	–
LPF	3 (1.9)	0 (0.00)	0.3
ACPRc	152 (98.1)	154 (99.4)	0.3
Total	155 (100)	155 (100)	

PCR corrected Adequate Clinical and Parasitological Response was study endpoint, ACPRc was not statistically different between DHA–PQ versus AL (p = 0.3)

^a^Non PCR corrected Adequate Clinical and Parasitological Response (ACPR), was statistically different between DHA–PQ versus AL

**Table 3:** Clinical and parasitological response at 42-day before and after PCR correction

Treatment arm outcome	AL n (%)	DHA–PQ n (%)	p-value
Before PCR correction			
LCF	9 (5.8)	4 (2.6)	0.15
LPF	32 (20.8)	5 (3.2)	< 0.0001
ACPR^a^	113 (73.4)	146 (94.2)	< 0.0001
Total	154 (100)	155 (100)	
After PCR correction			
LCF	0 (0.00)	1 (0.7)	1
LPF	4 (2.6)	0 (0.00)	0.06
ACPRc	150 (97.4)	154 (99.3)	0.2
Total	154 (100)	155 (100)	

ACPRc was not statistically different between DHA–PQ versus AL (p = 0.2)

^a^Non-PCR corrected Adequate Clinical and Parasitological Response was statistically different between DHA–PQ versus AL

### Electrocardiogram

Electrocardiogram was performed in a sub-group of 115 participants, and QTc values were measured before and after full dose of treatment. For technical reason because ECG machine broke down frequently we did not perform ECG for all patient that why QTc data analysis was performed for patient who had QTc value before and after treatment. This subset of participants was not chosen randomly.

The proportion of participants who had an increased QTc from baseline to 5 h after the last dose was similar between treatment arms 7% (95% CI 2.0–17.0) for AL versus 12.1% (95% CI 5.0–23.3) for DHA–PQ, p = 0.4), respectively as shown in Table 4. The mean QTc prolongation from baseline to day 3 was lower for DHA–PQ as well as for AL (4 ms and 8 ms) respectively as shown in Table 5.

**Table 4:** QTc normal and abnormal values between DHA–PQ and AL groups

QTc	Treatment arm		
	AL n (%)	DHA–PQ n (%)	p-value
Normal	53 (93.0)	51 (87.9)	–
Elevated	4 (7.0)	7 (12.1)	0.4
Total	57 (100)	58 (100)

**Table 5:** The mean QTc before and after treatment between DHA–PQ and AL groups

	Values at enrolment			Values at day 3 (5 h after last dose)		
	AL	DHA–PQ	p-value	AL	DHA–PQ	p-value
QTc mean (msec)	416.1	423.8	0.27	419.7	423.9	0.99
QTc SD (msec)	18.7	19.6	–	17.3	42.2	–

### Fever clearance and anaemia

**Fig. 3:**
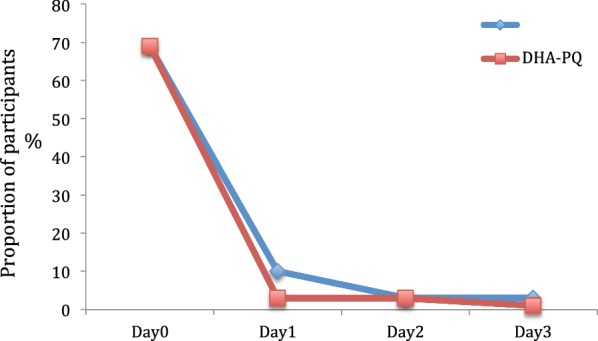
Proportion of participants with fever during 3 days after treatment initiation

As showed in Fig. 3, the proportion of participants who cleared the fever was similar on days 2 and 3 between the two arms. However, on day 1, the fever clearance rate was higher in DHA–PQ arm 97% versus 91% in AL arm (p = 0.02).

**Fig. 4:**
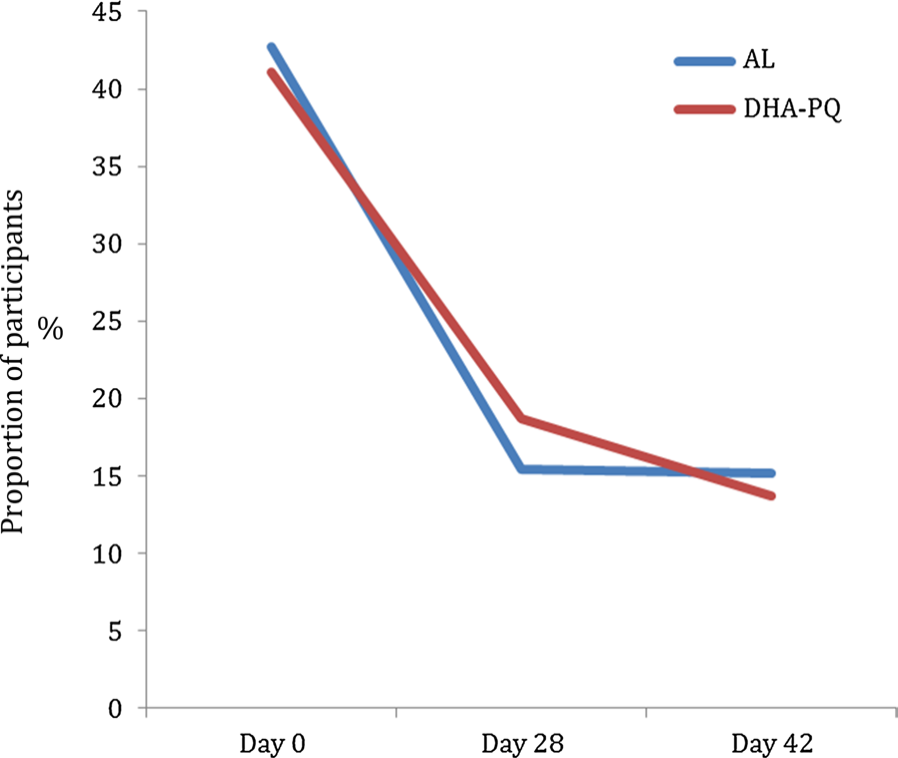
Clearance of anaemia between two study participants during follow-up. Anaemia was defined as a hemoglobin level < 10 g/dl

As showed in Fig. 4, the proportion of anaemia was similar at baseline (43% versus 41%) between DHA–PQ and AL groups as well as during the follow-up, 16% vs. 19% at day 28 (p = 0.5); 14% vs. 15% at day 42 (p = 0.8). Using McNemar paired test, both treatment arms significantly reduced the prevalence of anaemia at 28 and 42 day (p < 0.05).

## Discussion

The results of this study showed that DHA–PQ and AL drugs were efficacious in treating uncomplicated *P. falciparum* malaria in Doneguebougou and Torodo, Mali.

The parasite positivity rates of DHA–PQ and AL on day 2 and day 3 in our study were very similar to that find in a previous literature review and meta-analysis study [13, 14].

The PCR uncorrected cure rate at day 28 was significantly higher for DHA–PQ arm than AL cure rate 84.5%, (p < 0.001). These results are consistent with those reported previously in Zambia [7]. The day 28 PCR uncorrected cure rate was higher than that reported in multicentric trial achieved through Burkina Faso, Kenya, Mozambique, Uganda and Zambia [15]. For day 28 follow-up, 1.9% and 13.6% of participants had recurrent parasitaemia for DHA–PQ and AL arms, respectively, paired polymerase chain reaction (PCR) blots (from day 0 and the day of failure parasites) have been analysed for parasite merozoite surface protein genes 2 (*msp2*) and microsatellite (CA1 and TA99), to distinguish between re-infection and recrudescence as described previously in method section.

As stated in WHO guidelines for monitoring anti-malarial drug efficacy, data were analysed by two methods: the Kaplan–Meier method and per-protocol analysis. Theses two methods found that AL and DHA–PQ had similar survival rates.

The significantly lower risk of recurrent parasitaemia after treatment with DHA–PQ compared to AL is likely explained by differences in the elimination half-lives of the partner drugs. Piperaquine takes 2–3 weeks to be eliminated [16] compared to lumefantrine, which has an estimated elimination half-life of 4–10 days [17].

Fever clearance was statistically lower in DHA–PQ compared to AL 24 h after treatment initiation; but after 48 h both treatment arms had similar fever clearance rates for the rest of follow-up.

One study participant died after receiving one dose of study product (AL). Indeed, the parents removed their child from the study and gone for a traditional treatment. When the study physician in charge of treatment took the news of the child, the parents confirmed that the child was dead. The death of the child would not be related to the study product but rather because the treatment could not be administered for reasons of refusal. The occurence of death is nevertheless considered as a serious adverse event because the participant received at least one dose of the investigative product otherwise this case could be considered as a refusal case.

For technical reasons related to the ECG machine, QTc could not be measured in all participants at all times points. These results showed that 12.1% of participants who received DHA–PQ had QTc elevated versus 7% of elevated QTc in AL arm; there was no significant difference between both treatment arms, but the sample size was small for this subgroup ECG study. Previous study in Thai–Cambodia border showed a prolonged QTc interval in DHA–PQ arm compared to placebo arm [18]. Significant correlation between plasma piperaquine concentration and prolongation in QTc interval from baseline was displayed in the Thai–Cambodia study. Recent multicentric prospective and observational study in Burkina, Mozambique, Ghana and Tanzania reported that three patients had QTc higher than 500 ms after treatment with DHA–PQ [19]. The mean QTc prolongation from baseline to day 3 was lower for DHA–PQ as well for AL (4 ms and 8 ms) respectively. This finding is similar compared to the average of 3, 4 and 11 ms, found respectively in Burkina Faso, Mozambique and Tanzania in previous multicentric study [19]. The results and impact of other ACT on cardiotoxicity has been well documented in the literature but this is the first study that give safety data of Malacur^®^. QTc prolongation observed in this study was not clinically significant.

In the context of the emergence of Plasmodium resistance to artemisinin and derivatives in South-East Asia, it was necessary to continue to monitor the efficacy of ACT in Africa.

After more than one decade of use of AL and ASAQ combinations as a national first-line treatment policy for uncomplicated malaria cases in Mali. Furthermore, AL should be administered with a fatty meal for an effective absorption of lumefantrine. This study showed that the dihydroartemisinin–piperaquine combination had similar efficacy and tolerability as artemether–lumefantrine combination. The added value of this study is that DHA–PQ could be used as strengthen first-line treatment lines in Mali. As updated information, according WHO Malaria Policy Advisory Committee meeting held in September 2014, piperaquine dose should be increase, by day 42 follow up, the risk of recrudescence in patients receiving a piperaquine dose below 59 mg/kg was significantly higher compared to the patients receiving a higher dose [20].

## Conclusion

Before PCR correction, DHA–PQ ACT was more efficacious than AL to treat uncomplicated malaria in Malian patients. Both treatments have provided a similar QTc outcome. After PCR corrections both treatment shown similar efficacy.
